# Economics of botulinum toxin therapy: influence of the abobotulinumtoxinA package size on the costs of botulinum toxin therapy

**DOI:** 10.1186/s40734-017-0049-z

**Published:** 2017-04-27

**Authors:** Dirk Dressler, Fereshte Adib Saberi

**Affiliations:** Movement Disorders Section, Department of Neurology, Hannover Medical School, Carl-Neuberg-Str. 1, D-30625, Hannover, Germany

**Keywords:** AbobotulinumtoxinA, Package size, Economics, 300MU vial, 500MU vial, Botulinum toxin therapy, Dysport®

## Abstract

**Background:**

AbobotulinumtoxinA (Dysport®) was distributed for many years in vials containing 500MU (D500). Recently a new 300MU vial (D300) was additionally introduced (introduction). We wanted to explore whether more differentiated package sizes allow for more economic use of Dysport® in a large neurological botulinum toxin (BT) outpatient clinic.

**Methods:**

The study followed a retrospective chart review design based on our digital BT therapy data bank. All patients receiving Dysport® exclusively in a constant dose during the observation period (introduction ± 7 months) were included. Economic calculations are based on Dysport® prices as officially advertised in Germany. Sharing of vials between patients was not allowed.

**Results:**

Altogether 83 patients (51 with dystonia, 25 with spasticity, 3 with hemifacial spasm, 4 with other diagnoses) were included in this study. The total amount of BT used before and after introduction was 102525MU, the amount prescribed 138000MU and 116300MU (−21700MU, −15.7%), the costs €146103 and €125250 (−€ 20853, −14.3%). The price for D500 before and after introduction was €529.36, for D300 €339.71. The D500 price for 1MU before and after introduction is €1.0587, the D300 price for 1MU €1.1324 (+ €0.073, +7.0% against D500).

**Conclusions:**

More flexible packaging reduces drug costs for BT therapy considerably. Introducing smaller packaging sizes is technically possible and should be encouraged. Extra costs for registration and logistics are moderate. Further cost reductions may be possible by introduction of even smaller packaging sizes. They can be calculated based on our model.

## Background

Botulinum Toxin (BT) type A for therapeutic purposes is provided by several international manufacturers: Allergan (Dublin, Ireland) manufactures Botox® (onabotulinumtoxinA), Merz Pharmaceuticals (Frankfurt/M, Germany) Xeomin® (incobotulinumtoxinA) and Ipsen (Boulogne Bilancourt, France) Dysport® (abobotulinumtoxinA). BT type A drugs are sold as freeze-dried powders contained in vials [1]. Before application they need to be reconstituted with normal saline. BT content in the vials varies. The original content was 100MU for Botox® and Xeomin® and 500MU for Dysport® (D500). Over time package sizes were differentiated. Recently, an additional package size of 300MU was introduced for Dysport® (D300). We wanted to explore whether more differentiated package sizes allow for more economic use of Dysport® in a large neurologic BT outpatient clinic.

## Methods

### Setting

The study took place at the Movement Disorders Section of Hannover Medical School (HMS-MDS), Hannover, Germany. HMS-MDS is specialised in neurological BT therapy and attracts patients from the region and beyond. BT therapy is used in all neurological BT motor indications including dystonia, spasticity, infantile cerebral palsy, tremor and tics. It is also used in all hypersecretory indications of BT including hyperhidrosis, hypersalivation and hyperlacrimation. Other BT indications covered include chronic migraine as well as numerous special and experimental indications. BT therapy is used in registered indications as well as in in off-label indications. All three BT type A drugs are used. BT type B is used in special circumstances only. Annual BT consumption of HMS-MDS currently exceeds 12000 standard vials per year (1 standard vial = 1 vial Botox® 100MU = 1 vial Xeomin® 100MU = 1/3 vial Dysport® 500MU). Composition of patients treated is outlined in the results section and in Table 1.

**Table 1:** Composition of the patient base included in this study

Diagnosis	Patients				Dose
	number [*n*]	female [*n*]	male [*n*]	age mean ± SD [years]	mean ± SD [MU]
Dystonia	51	29	22	60.3 ± 13.5	499.5 ± 371.4
Cervical Dystonia	37	23	14		610.4 ± 371.4
Blepharospasm	10	6	4		189.0 ± 156.5
Bruxism	4	0	4		250.0 ± 115.5
Spasticity	25	12	15	56.7 ± 14.6	1180.0 ± 574.4
Arm Spasticity	16	7	9		985.0 ± 569.3
Hemispasticity	6	2	4		1341.7 ± 399.3
Paraspasticity	3	1	2		1600.0 ± 624.5
Tetraspasticity	2	2	0		1625.0 ± 671.8
Hemifacial Spasm	3	2	1	65.4 ± 16.0	43.3 ± 22.5
Others	4	4	0	48.1 ± 20.8	600.0 ± 294.4
Hyperhidrosis	2	2	0		500.0 ± 70.7
Stump Pain	1	1	0		300
Focal Dystonia	1	1	0		1000

### Definitions

Introduction was the day when D300 became available and was used at HMS-MDS, i.e. January 1^st^ 2014. Observation period was the time period from June 1^st^ 2013 (introduction minus 7 months) to July 30^th^ 2014 (introduction plus 7 months). Injection series is the set of BT injections given at one appointment. Interinjection interval is the time between two subsequent injection series.

### BT therapy

D500 is reconstituted with 5.0 ml 0.9% NaCl/H_2_O, D300 with 3.0 ml 0.9% NaCl/H_2_O. BT therapy is applied following international guidelines and the algorithms developed at HMS-MDS during the past decades. Most applications are performed under anatomical guidance. Where necessary, electromyography and ultrasound [2] is used for this purpose.

### Design

The design of the study followed a retrospective chart review design. All data evaluated were prospectively and continuously collected as part of our digital BT therapy data base. Data were retrieved using pre-programmed retrieval algorithms. During the observation period all patients of HMS-MDS fulfilling the inclusion criteria were included in this study. Inclusion criteria consisted of: (1) BT therapy with Dysport® exclusively during the observation period. (2) Constant Dysport® dose throughout the observation period. In eligible patients equal number of injection series before and after introduction were selected and used for further evaluation. Within the limits of the observation period either 1 or 2 injection series before and after introduction were selected.

### Economics

Economic calculations are based on Dysport® prices as officially advertised in Germany. They are inclusive of German value added tax (VAT) at currently 19%.

## Results

### Patients

Table 1 gives an overview about the patients’ demographic data and the composition of the patient base. Altogether 83 patients are included in this study. They reflect about 60% of all patients treated with Dysport® at HMS-MDS during the observation period. About 40% of all patients had to be excluded because of variable BT doses within the observation period and because of insufficient number of injection series. 51 patients (29 females, 22 males, age 60.3 ± 13.5 years) suffered from dystonia. 37 of them had cervical dystonia, 10 blepharospasm and 4 bruxism. 25 patients (12 females, 15 males, age 56.7 ± 14.6 years) suffered from spasticity. 16 of them had arm spasticity, 6 hemispasticity, 3 paraspasticity and 2 tetraspasticity. 3 patients were treated for hemifacial spasm (2 females, 1 male, age 65.4 ± 16.0 years). 4 patients (2 females, 2 males, age 48.1 ± 20.8 years) were treated for other diagnoses including hyperhidrosis (n = 2), stump pain (n = 1) and focal myokymia (n = 1).

### Economics

All economics data are shown in Table 2. The total amount of BT used before and after introduction was 102525MU. The identical figure reflects the study design. Before introduction the total amount of BT prescribed was 138000MU, after introduction it was 116300MU. With the introduction the total amount of BT prescribed was reduced by 21700MU (−15.7%). Before introduction there were 276 D500 prescribed, afterwards 175 D500 and 96 D300. The costs of the BT therapy were €146103 before introduction and €125250 afterwards. With the introduction the costs of BT therapy was reduced by €20853 (−14.3%). The price of D500 is €529.36, of D300 €339.71. The D500 price for 1MU is €1.0587. This price was not changed with the introduction. The D300 price for 1MU is €1.1324. This reflects a surcharge against the D500 price for 1MU of €0.073 (7.0%).

**Table 2:** Economic parameters of botulinum toxin therapy with Dysport® before and after introduction of an additional Dysport®300MU vial

Item	Before D300 introduction	After D300 introduction	Difference
D300 price [€]	n/a	339.71	n/a
D300 price per MU [€]	n/a	1.1324	n/a
D500 price [€]	529.36	529.36	0
D500 price per MU [€]	1.0587	1.0587	0
D300 price per MU vs D500 price per MU [€]	n/a	n/a	0.073 (7.0%)
total BT used [MU]	102525	102525	0
total BT prescribed [MU]	138000	116300	−21700 (−15.7%)
D500 prescribed [n]	276	175	−101 (−36.7%)
D300 prescribed [n]	nil	96	96 (+100%)
costs [€]	146103	125250	−20853 (−14.3%)
cost reduction in D500 price per MU [€]	n/a	n/a	−22974 (−15.7%)

## Discussion

BT therapy has some unique features not found in other pharmacological therapies:

1) BT therapy is costly. D500, an average dose for neurological indications, costs €529.36 in Germany. Although daily treatment costs calculated on an average interinjection interval of 90 days are €5.88 only, economic use of BT drugs is mandatory. 2) BT therapy is performed in numerous indication groups throughout numerous medical specialties and in a therapeutic dose range seen in no other drug [3, 4]. Whilst lowest doses in spasmodic dysphonia may be below Dysport® 10MU, highest doses used in wide-spread dystonia or spasticity may be as high as Dysport® 2000MU. The same is true for other BT type A drugs. This makes adequate pricing very difficult. Whilst Dysport® treatment for one patient with spasmodic dysphonia may cost less than €10.00, Dysport® treatment for one patient with wide-spread dystonia or spasticity may cost over €2000.00. This spread would be economically unjustifiable as costs for development, registration, marketing and distribution are identical in both conditions and actual drug production costs are negligible. Applying pure economic considerations would discriminate all patients with BT low dose indications. Finding an average price reflecting this enormous spread, however, is difficult. 3) Another specific feature of BT drugs is their biologically active ingredient, the botulinum neurotoxin. As it is extremely toxic therapeutic doses have to be extremely low. For example, the BNT content per D500 is 12.5 ng only [1]. This excessive low amount makes BNT susceptible to various physical interactions. Reducing BNT per vial has therefore been a long-term manufacturing challenge. Additionally, providing long-term stability data for registration of new package sizes is costly. 4) BT drugs are distributed as freeze-dried powder requiring reconstitution with 0.9% NaCl/H_2_0 before use. After reconstitution the shelf life of all BT drugs is limited to avoid potential bacterial contaminations and general decay. In most countries spread of the reconstituted vial amongst several patients is prohibited for legal (mostly reimbursement) reasons and for general hygienic considerations. In Germany this drug use is specified by the Arzneimittelverschreibungsverordnung (AMVV). Within the framework of economic, marketing and manufacturing challenges we wanted to study the economic effects of the D300 introduction. For this, we controlled interfering treatment variabilities by applying rigorous inclusion criteria. Generalisation of our findings and relevance for other BT therapy centres is based on the typical constitution of the patient pool treated at HMS-MDS including a mix of patients covering all major neurological indications including dystonia, spasticity, hemifacial spasm and others. With the exemption of few patients shown under ‘others’ all Dysport® applications followed indications registered in Germany.

In summary, D300 introduction reduced the costs of Dysport® by 14.3%. It would have reduced them by 15.7%, if the D300 price per MU would not have been increased against the D500 price per MU by 7%. Considering the additional costs for registration of D300 and more complicated logistics a surcharge of 7% seems moderate and adequate.

## Conclusions

More flexible packaging reduces drug costs for BT therapy considerably. Introduction of smaller packaging sizes is technically possible and should be encouraged. Extra costs for registration and more complicated logistics are moderate. Further cost reductions may be possible by introduction of even smaller packaging sizes. They can be calculated based on our model.

## Abbreviations

€: Euro
BT: Botulinum toxin
D300: Dysport® 300MU vial
D500: Dysport® 500MU vial
HMS-MDS: Hannover Medical School Movement Disorders Section
MU: Mouse units
VAT: Value added tax
